# Hoarding symptoms are associated with higher rates of disability than other medical and psychiatric disorders across multiple domains of functioning

**DOI:** 10.1186/s12888-022-04287-2

**Published:** 2022-10-15

**Authors:** Sara K. Nutley, Michael Read, Stephanie Martinez, Joseph Eichenbaum, Rachel L. Nosheny, Michael Weiner, R. Scott Mackin, Carol A. Mathews

**Affiliations:** 1 Department of Epidemiology, University of Florida, 2004 Mowry Road, Gainesville, FL 32610 USA; 2 Department of Psychiatry, University of Florida, 100 S Newell Drive, L4-100, Gainesville, FL 32610 USA; 3 San Francisco VA Medical Center, 4150 Clement St, San Francisco, CA 94121 USA; 4 Department of Radiology, University of California, San Francisco, 505 Parnassus Ave, San Francisco, CA 94143 USA; 5 Department of Psychiatry and Behavioral Sciences, University of California, San Francisco, 401 Parnassus Ave, San Francisco, CA 94143 USA

**Keywords:** Hoarding, Disability, Impairment, WHODAS, Activities of daily living, Major depressive disorder, Diabetes, Chronic pain

## Abstract

**Background:**

Hoarding symptoms are associated with functional impairment, though investigation of disability among individuals with hoarding disorder has largely focused on clutter-related impairment to home management activities and difficulties using space because of clutter. This analysis assesses disability among individuals with hoarding symptoms in multiple domains of everyday functioning, including cognition, mobility, self-care, interpersonal and community-level interactions, and home management. The magnitude of the association between hoarding and disability was compared to that of medical and psychiatric disorders with documented high disability burden, including major depressive disorder (MDD), diabetes, and chronic pain.

**Methods:**

Data were cross-sectionally collected from 16,312 adult participants enrolled in an internet-based research registry, the Brain Health Registry. Pearson’s chi-square tests and multivariable logistic regression models were used to quantify the relationship between hoarding and functional ability relative to MDD, diabetes, and chronic pain.

**Results:**

More than one in ten participants endorsed clinical (5.7%) or subclinical (5.7%) hoarding symptoms (CHS and SCHS, respectively). After adjusting for participant demographic characteristics and psychiatric and medical comorbidity, CHS and SCHS were associated with increased odds of impairment in all domains of functioning. Moderate to extreme impairment was endorsed more frequently by those with CHS or SCHS compared to those with self-reported MDD, diabetes, and/or chronic pain in nearly all domains (e.g., difficulty with day-to-day work or school: CHS: 18.7% vs. MDD: 11.8%, *p* < 0.0001) except mobility and self-care. While those with current depressive symptoms endorsed higher rates of impairment than those with hoarding symptoms, disability was most prevalent among those endorsing both hoarding and comorbid depressive symptoms.

**Conclusions:**

Hoarding symptoms are associated with profound disability in all domains of functioning. The burden of hoarding is comparable to that of other medical and psychiatric illnesses with known high rates of functional impairment. Future studies should examine the directionality and underlying causality of the observed associations, and possibly identify target interventions to minimize impairment associated with hoarding symptomatology.

**Supplementary Information:**

The online version contains supplementary material available at 10.1186/s12888-022-04287-2.

## Background

Disability has been broadly defined as impairment to one or more areas of functioning, including alterations in body structure or problems in body function, difficulty executing activities of daily living, and problems with involvement in everyday life [1]. A substantial proportion of individuals with medical and psychiatric health conditions experience disability, including those with cardiovascular disease, diabetes, chronic pain, major depressive disorder (MDD), and generalized anxiety disorder (GAD) [2–4]. For psychiatric conditions in particular, the negative functional impact of psychiatric symptoms is a necessary criterion for diagnosis. However, the relative impact of health conditions on disability varies widely and thus the identification of diseases associated with substantial functional impairment is crucial for clarifying objectives in national health policy, and for patient assessment and management [3, 5].

Hoarding disorder (HD) is a neuropsychiatric condition characterized by persistent difficulty discarding due to distress or indecision about what to discard. Though HD affects up to 6% of U.S. older adults and is associated with substantial reductions in overall health and quality of life [6–8], exploration of disability among those with HD is somewhat limited. As HD was previously conceptualized as a subtype of obsessive–compulsive disorder (OCD), studies investigating the impact of hoarding on functional ability have largely focused on hoarding behavior that occurs in the context of OCD and other anxiety disorders. In this population, hoarding symptoms have been associated with compromised functioning in social, familial, and occupational settings [9–20]. Even when adjusting for the effects of comorbid mood and anxiety disorders, as well as co-occurring OCD symptoms, increased hoarding symptom severity has been associated with limited activity involvement, financial dissatisfaction, and poor social adjustment [11, 17]. While some researchers have suggested that the impact of hoarding symptoms on disability may actually exceed that of co-occurring affective and obsessive–compulsive disorders [9], others have observed no difference in functioning between OCD patients with and without hoarding symptoms [16, 19].

Though hoarding symptoms may occur in the context of OCD, it is estimated that fewer than 20% of individuals with HD meet diagnostic criteria for OCD [8]. Despite evidence suggesting a clinical and etiologic distinction between HD and the hoarding symptoms in OCD [20, 21], only a small number of studies have expanded investigation of functional disability in hoarding to non-OCD populations. Studies assessing disability among individuals with HD commonly report increased difficulty with home management activities, including greater difficulty using space because of clutter [22–26]. However, impairment among those with HD has also been extended to social, occupational, and personal care settings [20, 23, 24, 27, 28]. As in the studies of OCD, some investigators have reported that the degree of impairment observed among individuals with HD may exceed that of individuals with other psychiatric conditions [27]. However, others have reported no difference in disability between these populations [20]. Despite some evidence linking hoarding to disability, investigation has been largely limited to small samples that often lack control populations and explore disability in a limited number of functional domains (i.e., home management, social, occupational, etc.). The full extent of the relationship between hoarding and functional ability is not clear, and the association between hoarding symptoms and disability relative to that of other medical and psychiatric conditions has not been fully described.

The purpose of this study was to explore global and hoarding-specific disability in a large sample of individuals with clinically relevant and subclinical hoarding symptoms using information collected from U.S. adults enrolled in an online research platform, the Brain Health Registry [29]. To assess the relative magnitude of the relationship between hoarding and disability, self-reported impairment among those with hoarding symptoms was compared to that of individuals with other debilitating medical and psychiatric conditions, including major depressive disorder, diabetes, and chronic pain. It was hypothesized that individuals with hoarding symptoms would endorse impairment in all domains of functioning, including interpersonal and community-level interaction (social), cognition, mobility, self-care, and home management. It was further hypothesized that the magnitude of the relationship between hoarding symptoms and disability would be greater than or equal to that of other health conditions associated with high disease burden.

## Methods

The Brain Health Registry (BHR) is an online research registry developed to advance research of brain aging and neuropsychiatric health. The platform is comprised of more than 70,000 U.S. adult participants who are semi-annually invited to complete neurocognitive assessments and self-report questionnaires evaluating medical and psychiatric health and health behaviors, including assessments of hoarding symptoms and functional ability [29]. BHR participants are recruited from all 50 U.S. states using a variety of recruitment sources, including owned (e.g., BHR website and press-releases), paid (e.g., online advertising, direct mail), and earned (e.g., word of mouth, publicity) media. The sole inclusion criteria for participation is age $\ge$ 18 years. Electric informed consent is obtained from all participants prior to enrollment in the research registry. Compared to the U.S general population, BHR participants are more likely to be female, White, of older age, and of higher educational attainment [7].

As of August 2020, a total of 16,312 BHR participants simultaneously completed assessments of hoarding symptoms, health history, and functional impairment, and were included in this secondary analysis. Though the BHR is longitudinal in nature and some participants completed all relevant assessments at multiple time points, this analysis uses data collected at participants’ most recent time point with complete data. All data points included in the final analysis were collected between January 2019 and August 2020.

### Demographic characteristics

Participants were asked to report their age (18–90; recoded as (1) less than 60 years and (2) 60 years or older), gender (male/female [only 2 gender identity options were available as options in the BHR at the time of data collection]), race (African American; Asian; White; other [includes Native American, Pacific Islander, other, and individuals who identified with more than one race; collapsed into a single category due to low endorsement]), ethnicity (Latinx/Hispanic; Not Latinx/Hispanic; Prefer not to say), and educational attainment (grammar school; high school; some college; two-year degree; four-year degree; master’s degree; doctoral degree; professional degree; recoded as (1) less than college degree, (2) 2- or 4-year college degree, and (3) graduate or professional degree). Self-reported height and weight were used to calculate body mass index (BMI). Participants were provided a list of height and weight ranges (e.g., 140–149 pounds) from which conservative BMI estimates were calculated (i.e., when exact BMI group was unclear based on reported height and weight, individuals were classified using the BMI category closest to “Normal Weight”; see Nutley et al. [7] for details).

### Hoarding

Current hoarding symptoms were assessed using an online adaptation of the Hoarding Rating Scale, Self-Report (HRS-SR), a 5-item assessment for the core features of hoarding disorder: clutter, difficulty discarding, excessive acquisition, and hoarding-related distress and impairment [30]. Individual items ask participants to rate the severity of hoarding symptoms using a 9-point Likert scale ranging from 0 (none) to 8 (extreme); items are summed for a total score ranging from 0 to 40, with higher scores indicating greater hoarding symptomatology. For all items, participants are instructed to answer with regard to how symptoms are currently (no specific timeframe specified). Previously validated for the screening of clinical hoarding among BHR participants [31], cutoffs of 10 and 14 have been used to identify individuals with subclinical (SCHS) and clinically relevant hoarding symptoms (CHS), respectively [6, 7]. Participants with total scores less than 10 were classified as having minimal or no hoarding symptoms (No HS).

### Functional impairment

The 12-item version of the World Health Organization Disability Assessment Scale 2.0 (WHODAS 2.0) was used to assess participants’ level of functioning in six major life domains including cognition (i.e., ability to concentrate and learn), mobility (i.e., ability to move around), self-care (i.e., ability to manage personal hygiene), getting along (i.e., ability to interact with others), life activities (i.e., ability to carry out responsibilities at home, work, and school), and participation in society (i.e., ability to engage in community) [32]. For each item participants are asked to rate the level of difficulty they have experienced while performing 12 activities of daily living over the past 30 days using a 5-point Likert scale ranging from 1 (no difficulty/none) to 5 (extreme difficulty/cannot do). For each item, a binary variable was created to identify individuals experiencing moderate to extreme difficulty with the respective task (i.e., scores were dichotomized for comparing individuals with scores between 3 and 5 to those with scores of 1 or 2).

A modified version of the Activities of Daily Living in Hoarding scale (ADL-H) was used to assess hoarding-related impairment in daily functioning [33]. Participants were asked to rate the level of difficulty they experience in performing seven daily living activities due to a hoarding or clutter problem. Individual items were rated from 1 (can do easily) to 5 (unable to do), with higher scores indicating greater hoarding-related impairment to daily functioning. Participants were further asked about the presence and severity of hoarding-related safety concerns, including queries related to fire hazards, blocked exits, and clutter outside the home. Individual items were rated from 1 (no problem) to 5 (severe problem), with higher scores indicating more severe safety concerns. For all items, a binary variable was created to identify individuals experiencing moderate or greater difficulty (or severity) with the respective task or safety concern (i.e., scores were dichotomized for comparing individuals with scores between 3 and 5 to those with scores of 1 or 2). In the case where individual items do not apply, a “not applicable” (NA) response was offered to participants; NA items were not considered in the development of binary indicators of impairment (i.e., marked as missing).

### Medical and psychiatric health

The association between hoarding symptoms and disability was compared to that of quantitatively assessed depressive symptoms and self-reported lifetime diagnoses of major depressive disorder (MDD), diabetes, and chronic pain – conditions that are recognized as leading causes of disability-adjusted life-years in the United States by the Global Burden of Disease (GBD) study (GBD equivalent and 2019 rank: MDD [GBD: Depressive Disorders, Rank: 9], diabetes [GBD: Diabetes, Rank: 5], chronic pain [GBD: Low Back Pain, Rank 3]). Current depressive symptom severity was assessed using the 9-item Patient Health Questionnaire (PHQ-9)[34]. For all items, participants were asked to report the frequency of depressive symptoms over the past two weeks using a 4-point Likert scale. Items were summed for a total score ranging from 0 to 27. Participants with total scores greater than 10 (i.e., moderate to severe depressive symptoms) were classified as having current depressive symptoms and those with scores of 10 or less were classified as having no or minimal depression [35]. As previously examined by Vieira Sordo et al. [36], self-reported lifetime diagnoses of MDD and diabetes were ascertained by asking participants if they have ever had or currently have the conditions of interest (yes/no) [36]. Participants were further queried about chronic pain using the following question: “Is chronic pain a problem for you?” (yes/no). Four separate categorical variables were used to describe comorbidity between hoarding symptoms, depressive symptoms, MDD, diabetes, and chronic pain. For all four variables, individuals were classified into one of four groups: (1) comorbid hoarding symptoms (CHS or SCHS) and the medical or psychiatric condition of interest (i.e., depression, diabetes, or chronic pain), (2) hoarding symptoms only (i.e., CHS or SCHS but no medical or psychiatric condition of interest), (3) medical or psychiatric condition of interest only (i.e., depression, diabetes, or chronic pain and no HS), or (4) neither hoarding symptoms nor the medical or psychiatric condition of interest (i.e., depression, diabetes, or chronic pain). For example, individuals were categorized into one of four groups when classifying the comorbidity between hoarding symptoms and MDD: (1) comorbid hoarding symptoms (CHS/SCHS) and MDD, (2) hoarding symptoms only, (3) MDD only, or (4) neither HS nor MDD.

To assess the association between hoarding and disability independent of co-occurring psychiatric and medical health conditions, variables quantifying psychiatric and medical disease burden were calculated by summing the number of comorbid health conditions endorsed by participants in each domain (i.e., psychiatric and medical). For psychiatric disease burden, participants were asked to report lifetime history of MDD, generalized anxiety disorder, specific or social phobia, panic disorder, posttraumatic stress disorder, OCD, eating disorder, drug or alcohol abuse, attention-deficit/hyperactivity disorder, autism, Tourette syndrome or Tourette disorder, chronic motor or vocal tic disorder, bipolar disorder, schizophrenia, and psychosis. For medical disease burden, participants were asked to report lifetime history of high blood pressure, high cholesterol, diabetes, heart disease, stroke, asthma, lung disease, arthritis, allergies, traumatic brain injury, concussion, sleep apnea, and chronic pain.

### Statistical analysis

The demographic and health characteristics of study participants with CHS, SCHS, and no HS were compared using Pearson’s chi-square tests and Kruskal–Wallis H tests. Pearson’s chi-square tests were then used to compare (1) the proportion of individuals with CHS, SCHS, and no HS who endorsed moderate to extreme impairment on each single item measure of global (WHODAS) or hoarding-related (ADL-H) disability, as well as (2) the proportion of individuals with hoarding symptoms and/or depressive symptoms, MDD, or chronic pain who endorsed moderate to extreme impairment on each measure of disability. For measures of global disability (i.e., WHODAS), separate, adjusted logistic regression models were used to quantify the association between hoarding symptoms and moderate to extreme functional impairment (i.e., 12 separate models predicting each single item measure of disability in the WHODAS). Models were adjusted to control for the effects of participant demographic (i.e., age, gender, race, and education) and health-related (i.e., BMI, co-occurring psychiatric burden, and co-occurring medical burden) characteristics. Of note, the Results section has been structured by type of disability assessment rather than type of statistical test (i.e., we first present results from all statistical tests/models assessing the relationship between hoarding and disability assessed via the WHODAS, and then results from tests corresponding to the ADL-H).

## Results

Approximately one-tenth of the study sample endorsed clinically relevant hoarding symptoms (11.4%). Of 16,312 BHR participants included in this analysis, 923 (5.7%) endorsed clinical hoarding behavior (i.e., HRS-SR total score > 14), 937 (5.7%) endorsed subclinical hoarding behavior (i.e., HRS-SR total score between 10 and 14), and 14,452 (88.6%) did not endorse hoarding symptoms (i.e., HRS-SR total score less than 10). A demographic overview of the sample is outlined in Table 1. On average, individuals with CHS and SCHS were younger than those without HS (Median age [IQR]: CHS: 63 [54, 70], SCHS: 63 [56, 70], No HS: 65 [57, 72], *p* < 0.001), slightly less likely to be White (CHS: 87.5%, SCHS: 86.5%, No HS: 91.4%, *p* < 0.001), and slightly less likely to have received college or graduate-level education (CHS: 78.3%, SCHS: 80.9%, No HS: 84.0%, *p* < 0.001). Additionally, those with CHS were more likely than those without HS to be female (78.3% vs. 73.3%, *p* < 0.001), though no difference in the distribution of gender was observed when comparing individuals with SCHS and those without HS. A small effect size was observed for all between-group differences in demographic characteristics.

**Table 1:** Overview of study sample, by hoarding symptoms

	**CHS** *N* = 923 (%)	**SCHS** *N* = 937 (%)	**No HS** *N* = 14,452 (%)	${\chi }^{2}$, p*	Effect Size^a^
**Gender**				11.5, 0.003	0.03
Male	200 (21.7)	247 (26.4)	3864 (26.7)		
Female	723 (78.3)	690 (73.6)	10,588 (73.3)		
**Race**				43.5, < 0.001	0.04
African American	23 (2.5)	21 (2.2)	240 (1.7)		
Asian	34 (3.7)	40 (4.3)	325 (2.3)		
Other or more than 1 race^b^	58 (6.3)	66 (7.0)	673 (4.7)		
White	808 (87.5)	810 (86.5)	13,214 (91.4)		
**Ethnicity**				3.1, 0.543	0.01
Hispanic/Latinx	25 (2.7)	36 (3.8)	441 (3.1)		
Not Hispanic/Latinx	884 (95.8)	888 (94.8)	13,835 (95.7)		
Prefer Not to Say	14 (1.5)	13 (1.4)	176 (1.2)		
**Education**				47.2, < 0.001	0.04
Grad/professional degree	319 (34.6)	379 (40.5)	6439 (44.6)		
College degree	404 (43.8)	379 (40.5)	5695 (39.4)		
Less than college	200 (21.7)	179 (19.1)	2318 (16.0)		
**BMI** ^c^				198.5, < 0.001	0.10
Normal	189 (32.9)	212 (36.6)	4695 (51.4)		
Overweight	160 (28.9)	191 (33.0)	2716 (29.8)		
Obese	225 (39.1)	176 (30.4)	1716 (18.8)		
**Age (median, IQR)**	63 (54, 70)	63 (56, 70)	65 (57, 72)	51.1, < 0.001	0.0
**Psychiatric disease burden** ^d^ (median, IQR)	1 (0,2)	1 (0, 2)	0 (0, 1)	568.4, < 0.001	0.03
**Medical disease burden** ^e^ (median, IQR)	4 (2,5)	3 (2, 5)	2 (1, 4)	331.2, < 0.001	0.02

*CHS* Clinically Relevant Hoarding Symptoms, *SCHS* Subclinical Hoarding Symptoms, *No HS* No Hoarding Symptoms, *IQR* Interquartile Range

^*^Chi-square test statistic and *p*-value from Pearson’s chi-square tests (Gender, Race, Ethnicity, Education, BMI) and Kruskal–Wallis H tests (age, psychiatric disease burden, medical disease burden)

^a^Estimates of effect size include Cramer’s V (Pearson’s chi-square test) and epsilon-squared (Kruskal Wallis H tests)

^b^Includes individuals who identified with Native American, Pacific Islander, or other, as well as individuals who identified with more than one race

^c^*N* = 10,280

^d^The number of comorbid psychiatric conditions endorsed by the participant, including generalized anxiety disorder, specific or social phobia, panic disorder, posttraumatic stress disorder, OCD, eating disorder, drug or alcohol abuse, attention-deficit/hyperactivity disorder, autism, Tourette syndrome or Tourette disorder, chronic motor or vocal tic disorder, bipolar disorder, schizophrenia, and psychosis

^e^The number of comorbid medical conditions endorsed by the participant, including high blood pressure, high cholesterol, diabetes, heart disease, stroke, asthma, lung disease, arthritis, allergies, traumatic brain injury, concussion, sleep apnea, and chronic pain

In terms of health history, those with CHS were more than twice as likely to report obesity than those without hoarding symptoms (39.1% vs. 18.8%, *p* < 0.001), and the psychiatric and medical disease burdens reported by those with hoarding were nearly double that of those without hoarding (Psychiatric burden: median = 2.0, IQR = [0,2] vs. median = 0, IQR = [0,1], *p* < 0.001; Medical burden: median = 4, IQR = [2, 5] vs. median = 2, IQR = [1, 4] *p* < 0.001). Rates of obesity among those with SCHS fell intermediate to those with and without hoarding, as did psychiatric and medical disease burden (all pairwise *p* < 0.001).

### Functional disability

**Fig. 1:**
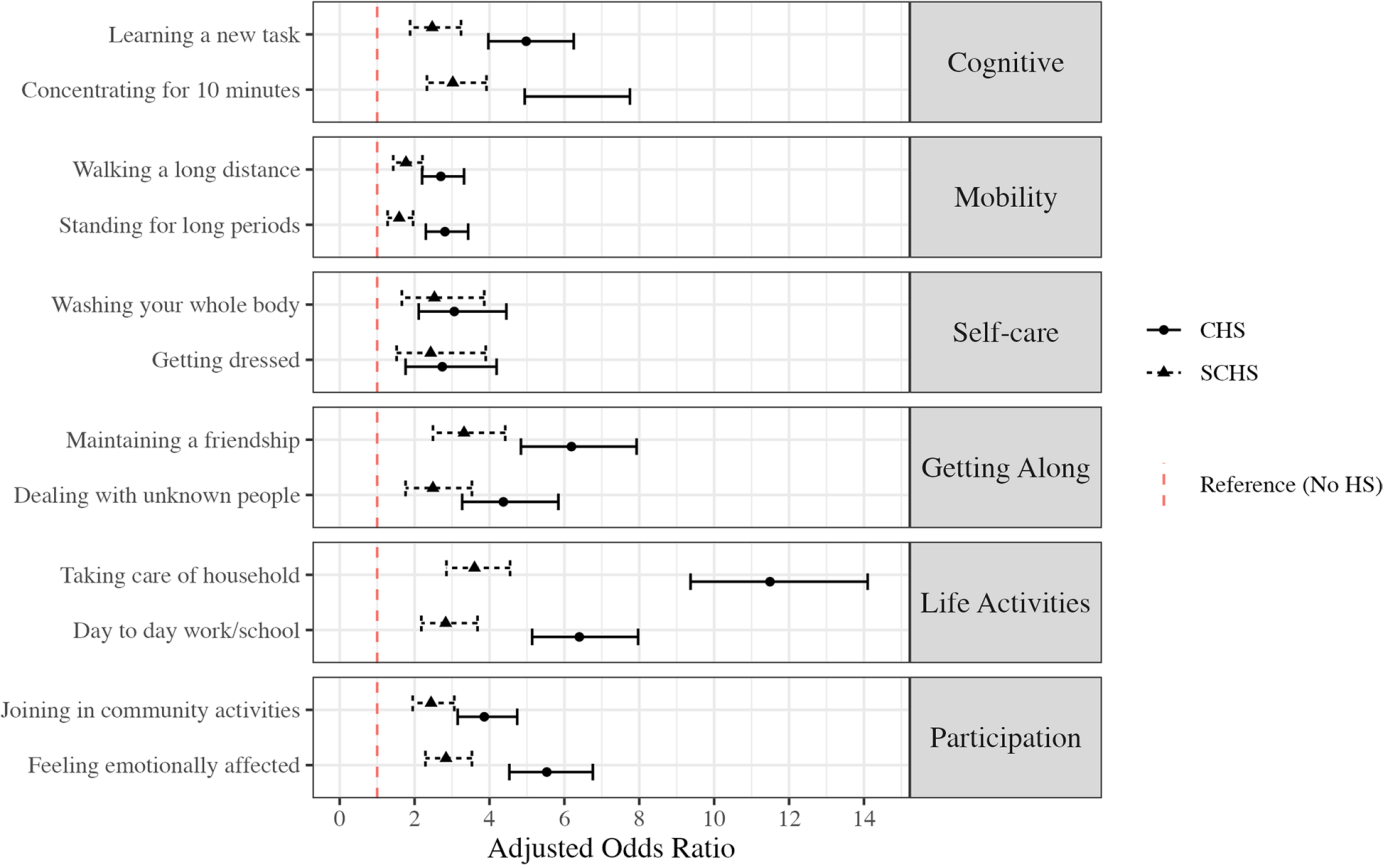
Separate adjusted logistic regression models predicting moderate-extreme disability in single-item WHODAS measures (Referent group: No Hoarding Symptoms). WHODAS: World Health Organization Disability Assessment Scale 2.0. CHS: Clinically relevant hoarding symptoms. SCHS: Subclinical hoarding symptoms. AOR: Adjusted Odds Ratio. 95% CI: 95% Confidence Interval. Referent group = No hoarding symptoms. Logistic regression models adjusted for demographic characteristics (i.e., gender, age, race, and education), body mass index, co-occurring psychiatric burden, and co-occurring medical burden

Moderate to extreme impairment was reported by more than one in three individuals with CHS for the majority of single item measures on the WHODAS, including measures of cognitive functioning, mobility, and life activities (Table 2). After adjusting for demographic characteristics and psychiatric/medical disease burden, CHS increased the odds of impairment in all domains. Notably, CHS was associated with a 6- to 12-fold increase in the odds of impairment in life activities/home management, as well as a 4- to sixfold increase in the odds of impairment in cognitive and social (i.e., interpersonal interaction, participation in society) functioning (Fig. 1; Table 3). Similarly, SCHS was associated with increased odds of impairment in social, physical (i.e., mobility), and cognitive domains relative to those without hoarding behavior (Fig. 3). However, impairment was less frequently reported by those with subclinical symptoms relative to those with CHS (Table 2); in adjusted regression models limited to participants with SCHS or CHS, individuals with CHS were between 1.5 and 3 times more likely than those with SCHS to endorse disability in all domains except self-care (Supplemental Table 1). Sensitivity analyses assessed whether functional impairment differed between individuals with and without hoarding symptoms when WHODAS items were measured using a full, 5-point Likert scale. For all measures of functional disability, individuals with CHS endorsed the highest levels of impairment, followed by those with SCHS, and those with no HS (data not shown). Bivariate Pearson correlation coefficients indicated that no single HRS item was uniquely associated with WHODAS summary scores (i.e., sum of all WHODAS items), rather that all HRS items were moderately correlated with disability, with correlation coefficients ranging from 0.35 to 0.44.

**Table 2:** Proportion endorsing of moderate-extreme impairment on WHODAS and ADL-H single item measures, by hoarding symptoms

**WHODAS item**	**CHS** *N* = 923 (%)	**SCHS** *N* = 937 (%)	**No HS** *N* = 14,452 (%)	${\chi }^{2}$, p*	Cramer’s V
Concentrating for 10 min	297 (32.3) ^a,b^	162 (17.3) ^a^	711 (4.9)	1123.8, < 0.001	0.26
Learning a new task	246 (26.7) ^a,b^	141 (15.1) ^a^	657 (4.6)	831.6, < 0.001	0.23
Standing for long periods	409 (44.3) ^a,b^	272 (29.0) ^a^	2196 (15.2)	595.0, < 0.001	0.19
Walking a long distance	387 (41.9) ^a,b^	275 (29.4) ^a^	2122 (14.7)	560.8, < 0.001	0.19
Washing your whole body	87 (9.4) ^a,b^	53 (5.7) ^a^	225 (1.6)	299.0, < 0.001	0.14
Getting dressed	72 (7.8) ^a,b^	45 (4.8) ^a^	185 (1.3)	250.5, < 0.001	0.12
Dealing with unknown people	155 (16.8) ^a,b^	95 (10.1) ^a^	385 (2.7)	566.5, < 0.001	0.19
Maintaining a friendship	232 (25.2) ^a,b^	124 (13.2) ^a^	467 (3.2)	1010.0, < 0.001	0.25
Taking care of household	478 (51.8) ^a,b^	209 (22.3) ^a^	779 (5.4)	2498.1, < 0.001	0.39
Day to day work/school	322 (34.9) ^a,b^	158 (16.9) ^a^	660 (4.6)	1374.7, < 0.001	0.29
Joining in community activities	354 (38.4) ^a,b^	222 (23.7) ^a^	1172 (8.1)	1004.2, < 0.001	0.25
Feeling emotionally affected	443 (48.1) ^a,b^	269 (28.7) ^a^	1339 (9.3)	1422.9, < 0.001	0.30
**ADL-H item**	**CHS** *N* = 923 (%)	**SCHS** *N* = 937 (%)	**No HS** *N* = 14,452 (%)	${\chi }^{2}$, p*	Cramer’s V
***Disability***					
Use stove	65 (7.2) ^a,b^	17 (1.8) ^a^	24 (0.2)	657.9, < 0.001	0.20
Use kitchen counters	239 (26.1) ^a,b^	79 (8.5) ^a^	96 (0.7)	2356.8, < 0.001	0.38
Eat at table	334 (37.6) ^a,b^	134 (14.7) ^a^	214 (1.5)	2911.9, < 0.001	0.43
Use bath/shower	51 (5.5) ^a,b^	11 (1.2) ^a^	30 (0.2)	439.7, < 0.001	0.17
Sit in sofa/chair	106 (11.5) ^a,b^	26 (2.8) ^a^	19 (0.1)	1245.9, < 0.001	0.28
Sleep in bed	75 (8.2) ^a,b^	22 (2.4) ^a^	26 (0.2)	760.2, < 0.001	0.22
Find important things	548 (59.6) ^a,b^	281 (30.0) ^a^	492 (3.4)	4252.4, < 0.001	0.51
***Safety***					
Fire hazard in the home	93 (10.1) ^a,b^	20 (2.1) ^a^	32 (0.2)	973.8, < 0.001	0.24
EMS ability to move through home	200 (21.7) ^a,b^	57 (6.1) ^a^	50 (0.4)	2231.1, < 0.001	0.37
Exits from home blocked	59 (6.4) ^a,b^	16 (1.7) ^a^	20 (0.1)	607.8, < 0.001	0.19
Difficulty moving up and down stairs	42 (4.6) ^a,b^	11 (1.2) ^a^	54 (0.4)	236.4, < 0.001	0.12
Clutter outside the home	173 (18.7) ^a,b^	68 (7.3) ^a^	127 (0.9)	1368.4, < 0.001	0.29

*CHS* Clinically Relevant Hoarding Symptoms, *SCHS* Subclinical Hoarding Symptoms, *No HS* No Hoarding Symptoms, *WHODAS* World Health Organization Disability Assessment Scale 2.0, *ADL-H* Activities of Daily Living in Hoarding

^*^Chi-square test statistic and *p*-value from Pearson’s chi-square tests

^a^significantly different from the no HS group (pairwise chi-square test, *p* < 0.01)

^b^significantly different from the SCHS group (pairwise chi-square test, *p* < 0.01)

**Table 3:** Separate adjusted logistic regression models predicting moderate-extreme disability in single-item WHODAS measures (Referent group: No Hoarding Symptoms)

	**CHS AOR** (95% CI)	**SCHS AOR** (95% CI)
Concentrating for 10 min	6.19 (4.94, 7.75)	3.02 (2.33, 3.92)
Learning a new task	4.98 (3.97, 6.25)	2.47 (1.88, 3.24)
Standing for long periods	2.81 (2.30, 3.43)	1.59 (1.28, 1.96)
Walking a long distance	2.70 (2.20, 3.32)	1.77 (1.43, 2.21)
Washing your whole body	3.06 (2.11, 4.45)	2.53 (1.66, 3.86)
Getting dressed	2.74 (1.76, 4.19)	2.43 (1.52, 3.90)
Dealing with unknown people	4.37 (3.27, 5.84)	2.49 (1.76, 3.53)
Maintaining a friendship	6.19 (4.84, 7.93)	3.32 (2.49, 4.42)
Taking care of household	11.49 (9.37, 14.10)	3.60 (2.85, 4.55)
Day to day work/school	6.40 (5.14, 7.97)	2.83 (2.18, 3.68)
Joining in community activities	3.86 (3.15, 4.74)	2.44 (1.95, 3.06)
Feeling emotionally affected	5.53 (4.53, 6.76)	2.84 (2.29, 3.53)

Referent group = No hoarding symptoms

*N* = 10,269–10,280

Logistic regression models adjusted for demographic characteristics (i.e., gender, age, race, and education), body mass index, co-occurring psychiatric burden, and co-occurring medical burden

*WHODAS* World Health Organization Disability Assessment Scale 2.0, *CHS* Clinically relevant hoarding symptoms, *SCHS* Subclinical hoarding symptoms, *AOR* Adjusted Odds Ratio, *95% CI* 95% Confidence Interval

### WHODAS: comparison to MDD, chronic pain, and diabetes

**Fig. 2:**
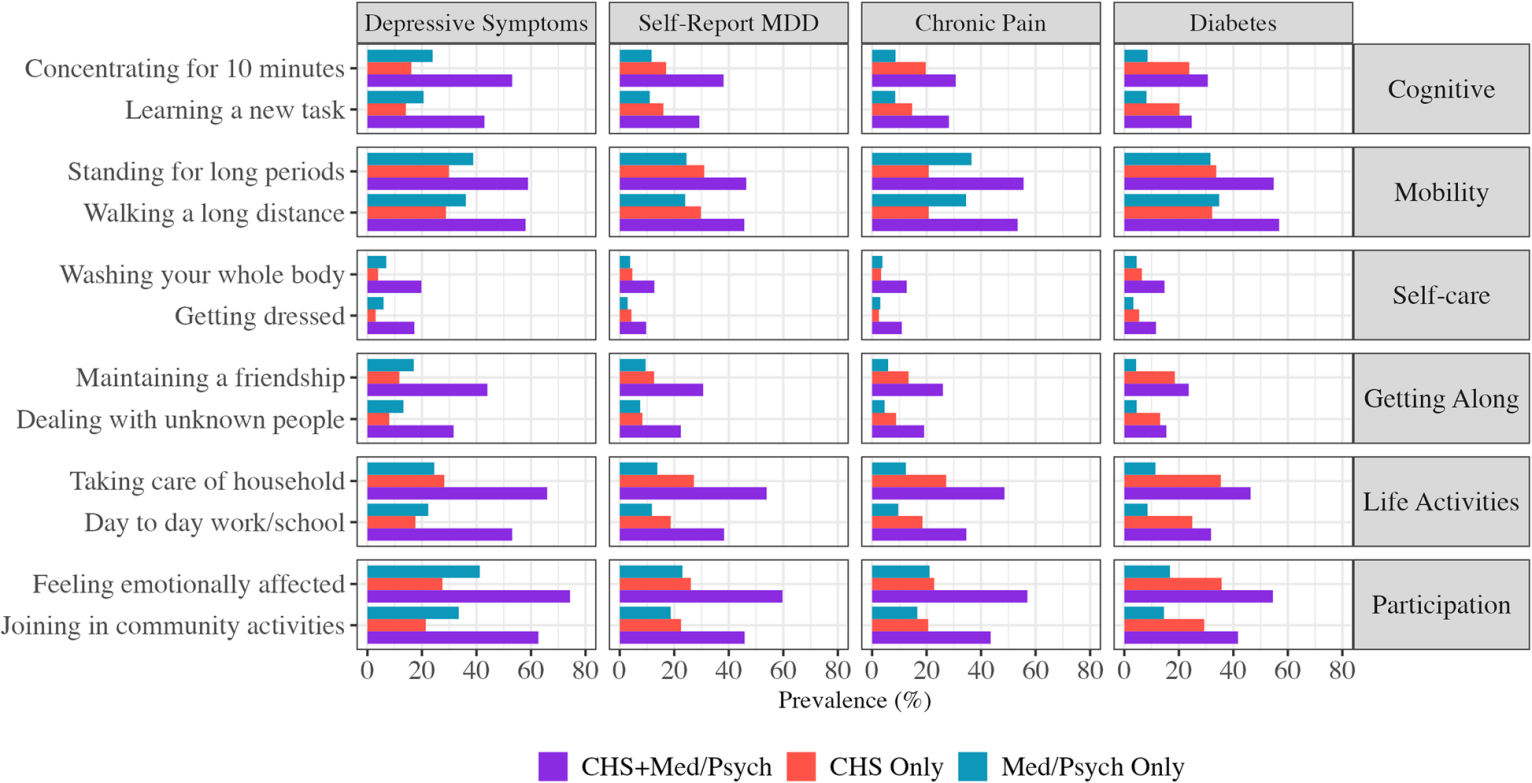
Prevalence of moderate-extreme impairment in WHODAS single item measures, by hoarding symptoms and medical/psychiatric comorbidity. WHODAS: World Health Organization Disability Assessment Scale 2.0. CHS: Clinically relevant hoarding symptoms. Med/Psych: Medical and psychiatric conditions of interest include (from left to right) depressive symptoms measured via the PHQ-9, major depressive disorder (MDD), chronic pain, and diabetes

The prevalence of moderate to extreme impairment in all single-item WHODAS measures for individuals with CHS, depressed mood, self-reported MDD, chronic pain, and diabetes are displayed in Fig. 2. When using self-report measures to assess lifetime history of MDD, chronic pain, and diabetes, individuals with CHS reported greater impairment in nearly all WHODAS domains (i.e., except mobility and self-care) than individuals with MDD only, those with chronic pain only, and those with diabetes only (Supplemental Table 2). However, the size of the association between CHS and disability was greatest when hoarding symptoms co-occurred with MDD, chronic pain, or diabetes. For all three conditions, all disease groups (i.e., CHS only, MDD/chronic pain only, CHS + MDD/chronic pain) reported significantly greater impairment than those with neither CHS nor MDD, chronic pain, or diabetes. A slightly different pattern was observed when using the PHQ-9 to assess current depressive symptoms. In all WHODAS domains, individuals with both CHS and current depressive symptoms most frequently reported moderate to extreme impairment, though individuals with depressed mood alone reported slightly greater impairment than those with CHS alone (Supplemental Table 2). However, in some domains, this difference between individuals with either CHS or depressive symptoms was marginal (e.g., life activities, getting along with others). Separate logistic regression models predicting moderate to extreme impairment in all single-item WHODAS measures using hoarding and depression severity scores as continuous indicators (i.e., HRS-SR and PHQ-9 total scores) led to similar results (Supplemental Table 3).

### Activities of daily living for hoarding disorder

As expected, very few individuals without HS reported impairment in activities of daily living (ADL) as a result of a clutter or hoarding problem. However, individuals with CHS frequently reported moderate to extreme difficulty in multiple domains, including being able to sit in a chair or sofa (11.5% vs. 0.2%, *p* < 0.0001), use kitchen counters (26.1% vs. 0.7%, *p* < 0.0001), eat at a table (37.6% vs. 1.5%, *p* < 0.0001), and find important things due to clutter or high volume of items in the home (59.6 vs. 3.4%, *p* < 0.0001; Table 2). Safety concerns were also common among those with CHS; approximately one-fifth of individuals with CHS reported concerns regarding emergency medical personnel’s ability to move equipment through their home (CHS:21.7% vs. No HS:0.4%, *p* < 0.0001), as well as concerns regarding excessive clutter outside their home, such as on a porch (18.7% vs. 0.9%, *p* < 0.0001). Additionally, nearly one in ten individuals with CHS reported an ongoing fire hazard in their home, though almost no participants without HS endorsed the same concern (10.1% vs. 0.2%, *p* < 0.0001). For all single item measures, the prevalence of safety concerns and impairment in common activities of daily living among those with SCHS were intermediate to those with and without clinically relevant hoarding symptoms (Table 2 [pairwise Pearson’s chi-square tests]). Findings remained when examining variability between hoarding groups when ADL-H items were measured using a 5-point Likert scale; for all single-item measures, individuals with CHS endorsed the highest levels of impairment, followed by those with SCHS, and those with no HS (data not shown).

### ADL-H: comparison to MDD, chronic pain, and diabetes

**Fig. 3:**
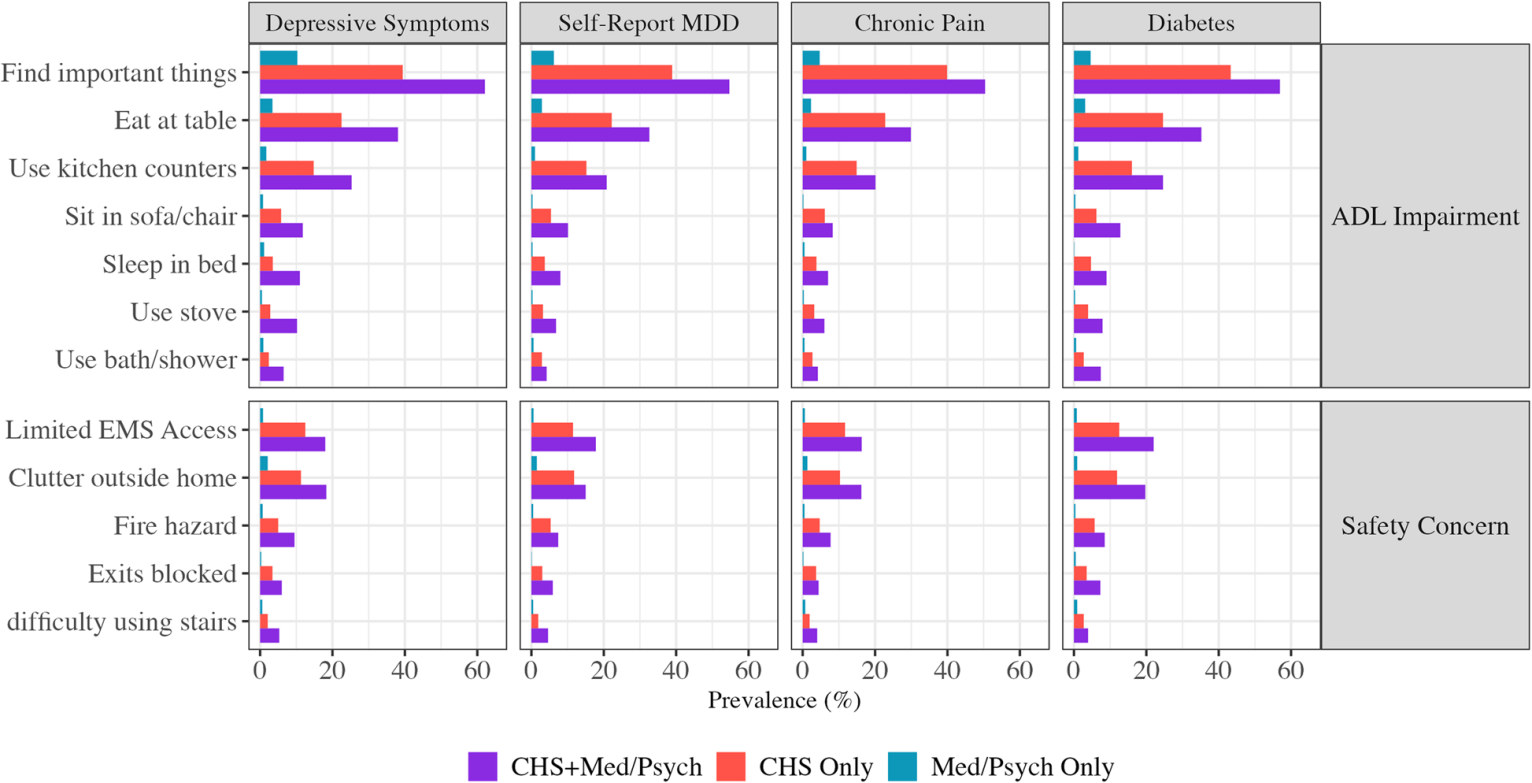
Prevalence of moderate-extreme impairment in ADL-H single item measures, by hoarding symptoms and medical/psychiatric comorbidity. ADL-H: Activities of Daily Living in Hoarding. CHS: Clinically relevant hoarding symptoms. Med/Psych: Medical and psychiatric conditions of interest include (from left to right) depressive symptoms measured via the PHQ-9, major depressive disorder

Despite the fact that clutter can also accumulate in the homes of individuals with active depression (e.g., due to amotivation), participants with current depressive symptoms (PHQ-9 scores > 10) but not CHS in this study rarely reported safety concerns or impairment to ADL due to a hoarding or clutter problem. The most frequently reported domain of ADL impairment among individuals with depressive symptoms only was difficulty finding important things (10.3%). However, those with CHS were almost four times as likely to report the same concern (39.4%, *p* < 0.001; Fig. 3; Supplemental Table 4). This effect was even stronger when assessing self-report diagnoses of MDD; those with CHS were over 6 times more likely than those with self-reported lifetime history of MDD to endorse difficulty finding important things as a result of a hoarding or clutter problem (38.9% vs. 6.2%, *p* < 0.0001). Regardless of how depressed mood was assessed, all ADL-H single item measures of disability and safety concerns were most frequently reported by those with both CHS and MDD/depressive symptoms, followed by those with CHS only, MDD only, and neither condition. The same pattern was observed when comparing individuals with CHS to those with chronic pain and those with diabetes (Fig. 3).

## Discussion

Although hoarding symptoms have previously been associated with functional impairment, to date much of the literature assessing disability among individuals with HD has focused on impairment to home management activities and difficulties using active living space because of clutter [22–26]. While our findings confirm that a substantial number of individuals with hoarding symptoms do experience difficulty in completing home tasks, our data indicate that impairment in other areas of functioning is also highly prevalent in those with hoarding symptoms. Perhaps most alarmingly, we observed a 2- to threefold increased odds of impairment in the domains of mobility and self-care among those with clinically significant hoarding symptoms or CHS, even after controlling for multiple possible confounders, as well as a 4 to sixfold increased odds of self-reported impaired cognitive and social functioning. We also observed high rates of disability among those with subclinical hoarding symptoms, though the size of the effect was slightly smaller than what we observed among those with CHS. Importantly, the observed associations occurred independently of co-occurring medical and psychiatric health burden, indicating that the relationship between disability and hoarding is not simply an attribute of high comorbidity rates.

As the relative impact of health conditions on disability varies widely, this analysis also uniquely assessed the magnitude of the association between hoarding symptoms and multidimensional functional impairment relative to medical and psychiatric conditions commonly associated with high disease burden: MDD, chronic pain, and diabetes. Our findings indicate that in nearly all domains of functioning, individuals with subclinical or clinical hoarding endorsed rates of impairment that were greater than or equal to that of individuals with self-reported depression, chronic pain, and/or diabetes, which are all ranked as among the top ten causes of disability in the U.S. by the WHO [37]. While rates of impairment among those with hoarding symptoms were slightly lower than what was observed among those with depressive symptoms, individuals with both hoarding and depressive symptoms were nearly twice as likely to report impairment in nearly all domains of functioning when compared to those with depression alone. As an estimated 50% of individuals with HD have comorbid MDD [8], the additive effect of hoarding and depressive symptoms on disability is of high concern. In this population, it is likely that concomitant treatment of HD and depressive symptoms is needed to improve overall functioning. However, HD remains vastly underdiagnosed and undertreated [38]. Assessment of hoarding symptoms among individuals with MDD who report substantial functional impairment may improve the identification of individuals with hoarding symptoms who may benefit from dual treatment programs.

Our findings further indicate that a substantial number of individuals with hoarding symptoms are at risk of house fires, falls, and other safety concerns due to hoarding behavior or clutter. Approximately one in ten individuals with CHS endorsed an ongoing fire-hazard in their home, and one in five endorsed concerns about emergency service personnel’s ability to access their home. Notably, these concerns were also present among individuals with subclinical hoarding. However, the self-reported prevalence of fire hazards in this sample was almost threefold lower than the rate observed in previous studies (~ 29%), and almost fivefold lower than the rate observed among community-dwelling cases identified through community agencies (~ 46%; [39]). It is important to note that the previously reported rates were based on direct observation, while our data rely on self-report. These discrepancies could be due to a lower severity of hoarding and clutter in our sample, which was unselected for HD, and thus may be more representative of the general population of individuals with clinically significant hoarding. However, they could also indicate that individuals with CHS may minimize the potential impact of their hoarding symptoms. Unfortunately, we do not have the ability within this study to determine which of these alternative explanations is more likely to be correct. Future studies comparing self-report to external observation would be needed.

As expected, individuals with clinical and subclinical hoarding symptoms also endorsed higher rates of impairment due to a hoarding or clutter problem than those with no or minimal hoarding symptoms. In part, this finding provides some evidence of the validity of the ADL-H assessment as a measure of hoarding-related impairment. However, our results also highlight the clinical relevance of distinguishing clinical and subclinical hoarding symptoms, as the two groups displayed differential rates of impairment to activities of daily living. Our findings indicate that while there are similarities between subclinical and clinical hoarding symptoms, there remain important distinctions regarding the impact of the disorder on overall functioning.

Several studies indicate improved functional ability among those with hoarding symptoms following treatment, though research has primarily focused on improvement in the domain of home management and ability to carry out other home tasks [40–47]. Few studies have expanded investigation to other areas of functioning, and data from these studies indicate little improvement in functional ability following treatment. For example, Ayers and colleagues (2011; [48]) found no evidence of improved functioning in social, home, or familial settings among older adults with hoarding following completion of an individual CBT program [48]. In part, this may be the result of relatively short follow-up periods that do not allow investigators to detect long-term changes in overall functioning. Additional investigation assessing the long-term impact of hoarding treatment on all domains of disability is needed. As our findings indicate that individuals with both hoarding symptoms and co-occurring depression or chronic pain are most likely to report impairment in all domains of functioning, attention to multimorbidity is likely a key component of effectively reducing disability among those with hoarding symptoms. Unique or combined intervention approaches may be needed for these populations.

Though not the primary focus of this analysis, our findings also indicated that individuals in the BHR sample who endorsed clinical and subclinical hoarding symptoms were slightly younger than those without hoarding symptoms. Though the size of the observed effect was small, this is a surprising observation given that the prevalence of hoarding has been found to increase linearly with age [49]. Additional investigation is needed to identify potential sources of the observed discrepancy.

To our knowledge, this is the first large-scale study to assess and compare disability in multiple domains of functioning among individuals with hoarding symptoms and those with MDD or chronic pain, which are conditions that are known to be associated with high disease burden. However, this investigation is not without limitations. Importantly, the use of cross-sectional data to assess the relationship between hoarding and disability precludes us from drawing definitive conclusions about the directionality of the observed association. This concern is compounded by use of self-report assessments that evaluate psychiatric symptomatology, health conditions, and disability using varying time frames, some of which allow for broad interpretation of time across the study sample (e.g., the HRS asks participants to answer with regard to how symptoms are currently). It is hypothesized that hoarding symptoms lead to increased impairment in overall functioning through interaction of multiple factors, including the accumulation of clutter within the home, increased social isolation, and financial distress. However, it is also possible that disability exacerbates hoarding symptoms and the accumulation of clutter. Additionally, it may be that an unknown factor simultaneously contributes to both hoarding symptoms and disability. Additional work is needed to explore the temporality of the association between hoarding and disability, as well as the underlying mechanisms of the observed relationship and potential confounding factors. If hoarding symptoms are found to precede functional impairment, it is possible that early recognition of hoarding behavior will lessen the functional impact of symptoms.

This investigation relies on the use of self-report measures to assess hoarding symptomatology and disability, introducing the possibility of self-report biases and some misclassification. Though previous investigation suggests that the use of the HRS-SR for identifying CHS and SCHS is a reasonable proxy for clinically-defined HD in the BHR sample, it is possible that social desirability biases or low insight contributed to artificially low levels of hoarding behavior for some individuals. It is also possible that some individuals with hoarding symptoms would be better classified using a diagnosis of a related disorder, such as OCD.

To assess the magnitude of the association between hoarding symptoms and disability relative to other conditions, the relationship between hoarding and functional impairment was compared to that of self-reported lifetime diagnoses of MDD, diabetes, and chronic pain. Though this comparison allows for a baseline understanding of the relative size of the observed association between hoarding and disability, some individuals reporting a lifetime history of medical and psychiatric conditions may not be currently affected, and thus the relationship between such conditions and disability is likely attenuated in our sample. Though we aimed to mitigate this concern by comparing the association between hoarding and disability to that of current depressive symptoms, additional work is needed to fully quantify the magnitude of the observed effect relative to other current medical and psychiatric symptoms. Lastly, the BHR inherently requires that individuals have computer and internet access for participation. As the sample is primarily comprised of White females of older age and high educational attainment, this investigation may not be representative of more diverse populations.

## Conclusions

Disability in hoarding is highly prevalent and extensive, affecting all areas of functioning including mobility, self-care, social and cognitive function, and home management. Impairment among those with clinical and subclinical hoarding symptoms is comparable to that of MDD and chronic pain, conditions known to be associated with high disability burden, and is further exacerbated by disease comorbidity. Efforts to reduce functional impairment in all domains, rather than focusing primarily on home-related domains are needed for those with hoarding; additional investigation should examine the role of current treatment programs in improving functional ability and work to identify specific approaches for targeting disability in all domains of functioning.

## Supplementary Information

**Additional file 1: Supplemental Table 1.** Separate adjusted logistic regression models predicting moderate-extreme disability in single-item WHODAS measures (Models limited to participants with hoarding symptoms; Referent group: Subclinical Hoarding Symptoms [SCHS]). **Supplemental Table 2.** Prevalence of moderate-extreme impairment in WHODAS single item measures, by hoarding symptoms and medical/psychiatric comorbidity. **Supplemental Table 3.** Odds of moderate-extreme disability corresponding to a single point increase in HRS and PHQ-9 total score. **Supplemental Table 4.** Prevalence of moderate-extreme impairment in ADL-H single item measures, by hoarding symptoms and medical/psychiatric comorbidity.

## Data Availability

The data that support the findings of this study are available from the Brain Health Registry following submission of a Data Sharing Request Form. Additional information is available at www.brainhealthregistry.org/for-investigators/de-identified-data-sharing.

## Abbreviations

MDD: Major depressive disorder
GAD: Generalized anxiety disorder
HD: Hoarding disorder
OCD: Obsessive compulsive disorder
BHR: Brain Health Registry
BMI: Body mass index
HRS-SR: Hoarding Rating Scale, Self-Report
SCHS: Subclinical hoarding symptoms
CHS: Clinical hoarding symptoms
No HS: No hoarding symptoms
WHODAS: World Health Organization Disability Assessment Scale
ADL-H: Activities of Daily Living in Hoarding
NA: Not applicable
GBD: Global Burden of Disease
PHQ-9: Patient Health Questionnaire (9-item)
IQR: Interquartile range
