# Supplementary material for: Hoarding symptoms are associated with higher rates of disability than other medical and psychiatric disorders across multiple domains of functioning
Source: BMC Psychiatry. 2022 Oct 15;22:647. doi: 10.1186/s12888-022-04287-2 (PMC9569124; doi:10.1186/s12888-022-04287-2)
Supplement: Supplementary file 1 — Additional file 1: Supplemental Table 1. Separate adjusted logistic regression models predicting moderate-extreme disability in single-item WHODAS measures (Models limited to participants with hoarding symptoms; Referent group: Subclinical Hoarding Symptoms [SCHS]). Supplemental Table 2. Prevalence of moderate-extreme impairment in WHODAS single item measures, by hoarding symptoms and medical/psychiatric comorbidity. Supplemental Table 3. Odds of moderate-extreme disability corresponding to a single point increase in HRS and PHQ-9 total score. Supplemental Table 4. Prevalence of moderate-extreme impairment in ADL-H single item measures, by hoarding symptoms and medical/psychiatric comorbidity. [file 12888_2022_4287_MOESM1_ESM.docx]

**Supplemental Table 1:** Separate adjusted logistic regression models predicting moderate-extreme disability in single-item WHODAS measures (Models limited to participants with hoarding symptoms; Referent group: Subclinical Hoarding Symptoms [SCHS])

|  | **CHS AOR**  (95% CI) |
| --- | --- |
| Concentrating for 10 minutes | 2.14 (1.58, 2.89) |
| Learning a new task | 2.13 (1.56, 2.91) |
| Standing for long periods | 1.81 (1.38, 2.37) |
| Walking a long distance | 1.54 (1.16, 2.04) |
| Washing your whole body | 1.22 (0.74, 1.99) |
| Getting dressed | 1.18 (0.69, 2.05) |
| Dealing with unknown people | 1.88 (1.26, 2.79) |
| Maintaining a friendship | 1.98 (1.42, 2.75) |
| Taking care of household | 3.17 (2.42, 4.15) |
| Day to day work/school | 2.34 (1.74, 3.14) |
| Joining in community activities | 1.64 (1.25. 2.17) |
| Feeling emotionally affected | 1.98 (1.51, 2.60) |

WHODAS: World Health Organization Disability Assessment Scale 2.0

CHS: Clinically relevant hoarding symptoms

SCHS: Subclinical hoarding symptoms

AOR: Adjusted Odds Ratio

95% CI: 95% Confidence Interval

Referent group = No hoarding symptoms

Logistic regression models adjusted for demographic characteristics (i.e., gender, age, race, and education), body mass index, co-occurring psychiatric burden, and co-occurring medical burden

**Supplemental Table 2:** Prevalence of moderate-extreme impairment in WHODAS single item measures, by hoarding symptoms and medical/psychiatric comorbidity

| **Self-Reported MDD** | | **CHS Only**  N=1181 (%) | **MDD Only**  N=2402 (%) | **CHS and MDD**  N=679 (%) | **Neither Condition**  N=12050 (%) | $\chi^{2}$, p* | Cramer’s V |
| --- | --- | --- | --- | --- | --- | --- | --- |
| Concentrating for 10 minutes | | 201 (17.0) | 282 (11.7) ^a^ | 258 (38.1) ^a,b^ | 429 (3.6) | 1456.4, <0.001 | 0.30 |
| Learning a new task | | 189 (16.0) | 264 (11.0) ^a^ | 198 (29.2) ^a,b^ | 393 (3.3) | 1051.3, <0.001 | 0.25 |
| Standing for long periods | | 366 (31.0) | 588 (24.5) ^a^ | 315 (46.4) ^a,b^ | 1608 (13.4) | 761.8, <0.001 | 0.22 |
| Walking a long distance | | 352 (29.8) | 577 (24.0) | 310 (45.7) ^a,b^ | 1545 (12.8) | 763.0, <0.001 | 0.22 |
| Washing your whole body | | 54 (4.6) | 90 (3.8) | 86 (12.7) ^a,b^ | 135 (1.1) | 460.7, <0.001 | 0.17 |
| Getting dressed | | 51 (4.3) | 69 (2.9) ^a^ | 66 (9.7) ^a,b^ | 116 (1.0) | 336.9, <0.001 | 0.14 |
| Dealing with unknown people | | 98 (8.3) | 179 (7.5) | 152 (22.4) ^a,b^ | 206 (1.7) | 916.6, <0.001 | 0.24 |
| Maintaining a friendship | | 148 (12.6) | 229 (9.5) ^a^ | 208 (30.6) ^a,b^ | 238 (2.0) | 1404.2, <0.001 | 0.29 |
| Taking care of household | | 321 (27.2) | 316 (13.8) ^a^ | 366 (53.9) ^a,b^ | 463 (3.8) | 2593.1, <0.001 | 0.40 |
| Day to day work/school | | 221 (18.7) | 282 (11.8) ^a^ | 259 (38.3) ^a,b^ | 378 (3.1) | 1624.4, <0.001 | 0.32 |
| Joining in community activities | | 265 (22.5) | 449 (18.7) ^a^ | 311 (45.8) ^a,b^ | 723 (6.0) | 1482.5, <0.001 | 0.30 |
| Feeling emotionally affected | | 307 (26.1) | 552 (23.0) | 405 (59.7) ^a,b^ | 787 (6.5) | 2199.1, <0.001 | 0.37 |
| **Depressive Symptoms (PHQ-9)** | **CHS Only**  N=1,429 (%) | | **Dep. Only**  N=1,762 (%) | **CHS and Dep.**  N=431 (%) | **Neither Condition**  N=12670 (%) | $\chi^{2}$, p* | Cramer’s V |
| Concentrating for 10 minutes | 230 (16.1) | | 421 (23.9) ^a^ | 229 (53.1) ^a,b^ | 290 (2.3) | 2733.7, <0.001 | 0.41 |
| Learning a new task | 202 (14.1) | | 362 (20.6) ^a^ | 185 (42.9) ^a,b^ | 295 (2.3) | 2043.7, <0.001 | 0.35 |
| Standing for long periods | 427 (29.9) | | 684 (38.8) ^a^ | 254 (58.9) ^a,b^ | 1512 (11.9) | 1483.3, <0.001 | 0.30 |
| Walking a long distance | 412 (28.8) | | 636 (36.1) ^a^ | 250 (58.0) ^a,b^ | 1486 (11.7) | 1358.5, <0.001 | 0.29 |
| Washing your whole body | 55 (3.9) | | 121 (6.9) ^a^ | 85 (19.8) ^a,b^ | 104 (0.8) | 910.1, <0.001 | 0.24 |
| Getting dressed | 43 (3.0) | | 103 (5.9) ^a^ | 74 (17.2) ^a,b^ | 82 (0.7) | 823.1, <0.001 | 0.22 |
| Dealing with unknown people | 114 (8.0) | | 232 (13.2) ^a^ | 136 (31.6) ^a,b^ | 153 (1.2) | 1594.9, <0.001 | 0.31 |
| Maintaining a friendship | 167 (11.7) | | 300 (17.0) ^a^ | 189 (44.0) ^a,b^ | 167 (1.3) | 2386.2, <0.001 | 0.38 |
| Taking care of household | 403 (28.2) | | 431 (24.5) | 284 (65.9) ^a,b^ | 348 (2.7) | 3471.0, <0.001 | 0.46 |
| Day to day work/school | 251 (17.6) | | 393 (22.3) ^a^ | 229 (53.1) ^a,b^ | 267 (2.1) | 2758.5, <0.001 | 0.41 |
| Joining in community activities | 306 (21.4) | | 589 (33.5) ^a^ | 270 (62.7) ^a,b^ | 583 (4.6) | 2832.6, <0.001 | 0.42 |
| Feeling emotionally affected | 392 (27.5) | | 725 (41.2) ^a^ | 320 (74.3) ^a,b^ | 614 (4.8) | 3778.5, <0.001 | 0.48 |
| **Self-Reported Pain** | **CHS Only**  N=1015 (%) | | **Pain Only**  N=3882 (%) | **CHS and Pain**  N=845 (%) | **Neither Condition**  N=10570 (%) | $\chi^{2}$, p* | Cramer’s V |
| Concentrating for 10 minutes | 200 (19.7) | | 334 (8.6) ^a^ | 259 (30.7) ^a,b^ | 377 (3.6) | 1159.9, <0.001 | 0.27 |
| Learning a new task | 149 (14.7) | | 331 (8.5) ^a^ | 238 (28.2) ^a,b^ | 326 (3.1) | 1007.6, <0.001 | 0.25 |
| Standing for long periods | 211 (20.8) | | 1418 (36.5) ^a^ | 470 (55.6) ^a,b^ | 778 (7.4) | 2567.7, <0.001 | 0.40 |
| Walking a long distance | 211 (20.8) | | 1339 (34.5) ^a^ | 451 (53.4) ^a,b^ | 783 (7.4) | 2335.8, <0.001 | 0.38 |
| Washing your whole body | 33 (3.3) | | 146 (3.8) | 107 (12.7) ^a,b^ | 79 (0.8) | 528.0, <0.001 | 0.19 |
| Getting dressed | 25 (2.5) | | 116 (3.0) | 92 (10.9) ^a,b^ | 69 (0.7) | 492.9, <0.001 | 0.17 |
| Dealing with unknown people | 89 (8.8) | | 177 (4.6) ^a^ | 161 (19.1) ^a,b^ | 208 (2.0) | 692.8, <0.001 | 0.21 |
| Maintaining a friendship | 136 (13.4) | | 228 (5.9) ^a^ | 220 (26.0) ^a,b^ | 239 (2.3) | 1101.7, <0.001 | 0.26 |
| Taking care of household | 276 (27.2) | | 482 (12.4) ^a^ | 411 (48.6) ^a,b^ | 297 (2.8) | 2583.7, <0.001 | 0.40 |
| Day to day work/school | 188 (18.5) | | 373 (9.6) ^a^ | 292 (34.6) ^a,b^ | 287 (2.7) | 1533.6, <0.001 | 0.31 |
| Joining in community activities | 209 (20.6) | | 642 (16.6) ^a^ | 367 (43.5) ^a,b^ | 530 (5.0) | 1546.9, <0.001 | 0.31 |
| Feeling emotionally affected | 231 (22.8) | | 817 (21.1) | 481 (57.0) ^a,b^ | 522 (4.9) | 2423.6, <0.001 | 0.39 |
| **Diabetes** | **CHS Only**  N=1600 (%) | | **Diabetes Only**  N=1003 (%) | **CHS and Diabetes**  N=258 (%) | **Neither Condition**  N=13449 (%) | $\chi^{2}$, p* | Cramer’s V |
| Concentrating for 10 minutes | 380 (23.8) | | 85 (8.5) ^a^ | 79 (30.6) ^b^ | 626 (4.7) | 1003.9, <0.001 | 0.25 |
| Learning a new task | 323 (20.2) | | 81 (8.1) ^a^ | 64 (24.7) ^b^ | 576 (4.3) | 757.2, <0.001 | 0.22 |
| Standing for long periods | 539 (33.7) | | 317 (31.6) | 142 (54.8) ^a,b^ | 1879 (14.0) | 788.7, <0.001 | 0.22 |
| Walking a long distance | 515 (32.2) | | 349 (34.8) | 147 (56.8) ^a,b^ | 1773 (13.2) | 912.7, <0.001 | 0.24 |
| Washing your whole body | 102 (6.4) | | 45 (4.5) | 38 (14.7) ^a,b^ | 180 (1.3) | 381.1, <0.001 | 0.15 |
| Getting dressed | 87 (5.4) | | 33 (3.3) | 30 (11.6) ^a,b^ | 152 (1.1) | 298.0, <0.001 | 0.14 |
| Dealing with unknown people | 210 (13.1) | | 45 (4.5) ^a^ | 40 (15.4) ^b^ | 340 (2.5) | 524.2, <0.001 | 0.18 |
| Maintaining a friendship | 295 (18.5) | | 43 (4.3) ^a^ | 61 (23.6) ^b^ | 424 (3.2) | 886.0, <0.001 | 0.23 |
| Taking care of household | 567 (35.4) | | 114 (11.4) ^a^ | 120 (46.3) ^a,b^ | 665 (5.0) | 2083.6, <0.001 | 0.36 |
| Day to day work/school | 398 (24.9) | | 85 (8.5) ^a^ | 82 (31.8) ^b^ | 575 (4.3) | 1185.3, <0.001 | 0.27 |
| Joining in community activities | 468 (29.3) | | 145 (14.5) ^a^ | 108 (41.7) ^a,b^ | 1027 (7.6) | 981.6, <0.001 | 0.25 |
| Feeling emotionally affected | 571 (35.7) | | 167 (16.7) ^a^ | 141 (54.4) ^a,b^ | 1172 (8.7) | 1389.1, <0.001 | 0.29 |

WHODAS: World Health Organization Disability Assessment Scale 2.0

CHS: Clinically relevant hoarding symptoms

Dep.: Depressive Symptoms measured via the PHQ-9

* Chi-square test statistic and p-value from Pearson’s chi-square tests

^a^ significantly different from the ‘CHS only’ group (pairwise chi-square test, p<0.01)

^b^ significantly different from the ‘[medical/psychiatric condition] only’ group (pairwise chi-square test, p<0.01)

**Supplemental Table 3:** Odds of moderate-extreme disability corresponding to a single point increase in HRS and PHQ-9 total score

|  | **HRS AOR**  (95% CI) | **PHQ-9 AOR**  (95% CI) |
| --- | --- | --- |
| Concentrating for 10 minutes | 1.07 (1.06, 1.08) | 1.23 (1.21, 1.24) |
| Learning a new task | 1.05 (1.04, 1.06) | 1.19 (1.18, 1.20) |
| Standing for long periods | 1.04 (1.04, 1.05) | 1.13 (1.13, 1.14) |
| Walking a long distance | 1.04 (1.03, 1.05) | 1.13 (1.12, 1.14) |
| Washing your whole body | 1.04 (1.03, 1.07) | 1.16 (1.15, 1.18) |
| Getting dressed | 1.04 (1.02, 1.05) | 1.17 (1.15, 1.19) |
| Dealing with unknown people | 1.05 (1.06, 1.08) | 1.20 (1.18, 1.21) |
| Maintaining a friendship | 1.07 (1.06, 1.08) | 1.21 (1.20, 1.22) |
| Taking care of household | 1.13 (1.12, 1.14) | 1.19 (1.18, 1.21) |
| Day to day work/school | 1.08 (1.07, 1.09) | 1.21 (1.20, 1.23) |
| Joining in community activities | 1.05 (1.04, 1.06) | 1.21 (1.20, 1.23) |
| Feeling emotionally affected | 1.07 (1.06, 1.08) | 1.25 (1.24, 1.27) |

12 multiple logistic regression models predicting moderate-extreme disability in single-item WHODAS measures using HRS-SR and PHQ-9 total scores as predictor variables

WHODAS: World Health Organization Disability Assessment Scale 2.0

HRS: Hoarding Rating Scale

PHQ-9: 9-item Patient Health Questionnaire

AOR: Adjusted Odds Ratio (ORs adjusted for PHQ-9 total score and HRS-SR total score, respectively)

95% CI: 95% Confidence Interval

N=10,269-10,280

**Supplemental Table 4:** Prevalence of moderate-extreme impairment in ADL-H single item measures, by hoarding symptoms and medical/psychiatric comorbidity

| **Self-Reported MDD** | **CHS Only**  N=1181 | **MDD Only**  N=2402 | **CHS and MDD**  N=679 | **Neither Cond.**  N=12050 | $\chi^{2}$, p* | Cramer’s V |
| --- | --- | --- | --- | --- | --- | --- |
| ***Disability*** |  |  |  |  |  |  |
| Use stove | 37 (3.2) | 7 (0.3) ^a^ | 45 (6.8) ^a,b^ | 17 (0.1) | 542.5, <0.001 | 0.18 |
| Use kitchen counters | 178 (15.2) | 24 (1.0) ^a^ | 140 (20.8) ^a,b^ | 0.6 () | 1842.4, <0.001 | 0.34 |
| Eat at table | 254 (22.2) | 67 (2.9) ^a^ | 214 (32.6) ^a,b^ | 147 (1.2) | 2461.7, <0.001 | 0.39 |
| Use bath/shower | 34 (2.9) | 13 (0.6) ^a^ | 28 (4.2) ^b^ | 17 (0.1) | 302.2, <0.001 | 0.14 |
| Sit in sofa/chair | 64 (5.4) | 8 (0.3) ^a^ | 68 (10.1) ^a,b^ | 11 (0.1) | 966.0, <0.001 | 0.24 |
| Sleep in bed | 43 (3.7) | 8 (0.3) ^a^ | 54 (8.0) ^a,b^ | 18 (0.2) | 663.0, <0.001 | 0.20 |
| Find important things | 458 (38.9) | 147 (6.2) ^a^ | 371 (54.7) ^a,b^ | 354 (2.9) | 3885.4, <0.001 | 0.49 |
| ***Safety Concerns*** |  |  |  |  |  |  |
| Fire hazard in the home | 63 (5.3) | 11 (0.5) ^a^ | 50 (7.4) ^b^ | 21 (0.2) | 662.9, <0.001 | 0.20 |
| EMS ability to move through home | 136 (11.5) | 15 (0.6) ^a^ | 121 (17.8) ^a,b^ | 35 (0.3) | 1713.5, <0.001 | 0.32 |
| Exits from home blocked | 35 (3.0) | 2 (0.1) ^a^ | 40 (5.9) ^a,b^ | 18 (0.2) | 495.5, <0.001 | 0.17 |
| Difficulty moving up and down stairs | 22 (1.9) | 12 (0.5) ^a^ | 31 (4.6) ^a,b^ | 42 (0.4) | 204.0, <0.001 | 0.11 |
| Clutter outside the home | 139 (11.8) | 37 (1.5) ^a^ | 102 (15.0) ^b^ | 90 (0.8) | 116.6, <0.0001 | 0.26 |
| **Depressive Symptoms** | **CHS Only**  N=1429 | **Dep. Only**  N=1762 | **CHS and Dep.**  N=431 | **Neither Cond.**  N=12670 | $\chi^{2}$, p* | Cramer’s V |
| ***Disability*** |  |  |  |  |  |  |
| Use stove | 39 (2.8) | 8 (0.5) ^a^ | 43 (10.2) ^a,b^ | 16 (0.1) | 733.6, <0.001 | 0.21 |
| Use kitchen counters | 210 (14.8) | 29 (1.7) ^a^ | 108 (25.3) ^a,b^ | 67 (0.5) | 1940.1, <0.001 | 0.35 |
| Eat at table | 322 (22.5) | 58 (3.4) ^a^ | 156 (38.1) ^a,b^ | 156 (1.3) | 2543.8, <0.001 | 0.40 |
| Use bath/shower | 34 (2.4) | 15 (0.9) ^a^ | 28 (6.5) ^a,b^ | 15 (0.1) | 399.5, <0.001 | 0.16 |
| Sit in sofa/chair | 82 (5.8) | 13 (0.8) ^a^ | 50 (11.8) ^a,b^ | 6 (0.1) | 1001.7, <0.001 | 0.25 |
| Sleep in bed | 50 (3.5) | 19 (1.1) ^a^ | 47 (11.0) ^a,b^ | 7 (0.1) | 819.4, <0.001 | 0.23 |
| Find important things | 562 (39.4) | 179 (10.3) ^a^ | 267 (62.1) ^a,b^ | 313 (2.5) | 4063.4, <0.001 | 0.50 |
| ***Safety Concerns*** |  |  |  |  |  |  |
| Fire hazard in the home | 72 (5.0) | 13 (0.7) ^a^ | 41 (9.5) ^a,b^ | 19 (0.2) | 722.3, <0.001 | 0.21 |
| EMS ability to move through home | 179 (12.5) | 14 (0.8) ^a^ | 78 (18.0) ^a,b^ | 36 (0.3) | 1677.3, <0.001 | 0.32 |
| Exits from home blocked | 49 (3.4) | 5 (0.3) ^a^ | 26 (6.0) ^b^ | 15 (0.1) | 471.0, <0.001 | 0.17 |
| Difficulty moving up and down stairs | 30 (2.1) | 10 (0.6) ^a^ | 23 (5.3) ^a,b^ | 44 (0.4) | 209.4, <0.001 | 0.11 |
| Clutter outside the home | 162 (11.3) | 37 (2.1) ^a^ | 79 (18.3) ^a,b^ | 90 (0.7) | 1177.2, <0.001 | 0.27 |
| **Self-Reported Pain** | **CHS Only**  N=1015 | **Pain Only**  N=3882 | **CHS and Pain**  N=845 | **Neither Cond.**  N=10570 | $\chi^{2}$, p* | Cramer’s V |
| ***Disability*** |  |  |  |  |  |  |
| Use stove | 32 (3.2) | 11 (0.3) ^a^ | 50 (6.0) ^a,b^ | 13 (0.1) | 514.7, <0.001 | 0.18 |
| Use kitchen counters | 150 (14.9) | 40 (1.0) ^a^ | 168 (20.1) ^a,b^ | 56 (0.5) | 1840.3, <0.001 | 0.34 |
| Eat at table | 224 (22.8) | 86 (2.3) ^a^ | 244 (29.9) ^a,b^ | 128 (1.2) | 2402.3, <0.001 | 0.39 |
| Use bath/shower | 27 (2.7) | 18 (0.5) ^a^ | 35 (4.2) ^b^ | 12 (0.1) | 309.2, <0.001 | 0.14 |
| Sit in sofa/chair | 62 (6.1) | 8 (0.2) ^a^ | 70 (8.3) ^b^ | 11 (0.1) | 889.5, <0.001 | 0.23 |
| Sleep in bed | 38 (3.8) | 18 (0.5) ^a^ | 59 (7.0) ^a,b^ | 8 (0.1) | 625.1, <0.001 | 0.20 |
| Find important things | 404 (39.9) | 182 (4.7) ^a^ | 425 (50.4) ^a,b^ | 310 (3.0) | 3791.8, <0.001 | 0.48 |
| ***Safety Concerns*** |  |  |  |  |  |  |
| Fire hazard in the home | 48 (4.7) | 21 (0.5) ^a^ | 65 (7.7) ^a,b^ | 11 (0.1) | 693.1, <0.001 | 0.21 |
| EMS ability to move through home | 119 (11.7) | 22 (0.6) ^a^ | 138 (16.3) ^a,b^ | 28 (0.3) | 1673.9, <0.001 | 0.32 |
| Exits from home blocked | 38 (3.7) | 6 (0.2) ^a^ | 37 (4.4) ^b^ | 14 (0.1) | 434.8, <0.001 | 0.16 |
| Difficulty moving up and down stairs | 19 (1.9) | 28 (0.7) ^a^ | 34 (4.0) ^a,b^ | 26 (0.3) | 197.6, <0.001 | 0.11 |
| Clutter outside the home | 104 (10.3) | 51 (1.3) ^a^ | 137 (16.2) ^a,b^ | 76 (0.7) | 1169.2, <0.001 | 0.27 |
| **Diabetes** | **CHS Only**  N=1600 | **Diabetes Only**  N=1003 | **CHS and Diabetes**  N=258 | **Neither Cond.**  N=13449 | $\chi^{2}$, p* | Cramer’s V |
| ***Disability*** |  |  |  |  |  |  |
| Use stove | 62 (3.9) | 3 (0.3) ^a^ | 20 (7.9) ^a,b^ | 21 (0.2) | 511.3, <0.001 | 0.18 |
| Use kitchen counters | 255 (16.0) | 12 (1.2) ^a^ | 63 (24.6) ^a,b^ | 84 (0.6) | 1853.7, <0.001 | 0.34 |
| Eat at table | 381 (24.6) | 30 (3.1) ^a^ | 87 (35.2) ^a,b^ | 184 (1.4) | 2404.5, <0.001 | 0.39 |
| Use bath/shower | 43 (2.7) | 6 (0.6) ^a^ | 19 (7.4) ^a,b^ | 24 (0.2) | 374.3, <0.001 | 0.15 |
| Sit in sofa/chair | 99 (6.2) | 4 (0.4) ^a^ | 33 (12.8) ^a,b^ | 15 (0.11) | 970.6, <0.001 | 0.25 |
| Sleep in bed | 74 (4.7) | 1 (0.1) ^a^ | 23 (9.0) ^a,b^ | 25 (0.2) | 608.3, <0.001 | 0.19 |
| Find important things | 693 (43.3) | 46 (4.6) ^a^ | 136 (56.9) ^a,b^ | 446 (3.4) | 3742.6, <0.001 | 0.48 |
| ***Safety Concerns*** |  |  |  |  |  |  |
| Fire hazard in the home | 91 (5.7) | 4 (0.4) ^a^ | 22 (8.5) ^b^ | 28 (0.2) | 661.3, <0.001 | 0.20 |
| EMS ability to move through home | 200 (12.5) | 8 (0.8) ^a^ | 57 (22.0) ^a,b^ | 42 (0.3) | 1730.0, <0.001 | 0.33 |
| Exits from home blocked | 56 (3.5) | 5 (0.5) ^a^ | 19 (7.3) ^a,b^ | 15 (0.1) | 490.7, <0.001 | 0.17 |
| Difficulty moving up and down stairs | 43 (2.7) | 9 (0.9) ^a^ | 10 (3.9) ^b^ | 45 (0.3) | 164.3, <0.001 | 0.10 |
| Clutter outside the home | 190 (11.9) | 9 (0.9) ^a^ | 51 (19.7) ^a,b^ | 118 (0.9) | 1152.1, <0.001 | 0.27 |

ADL-H: Activities of Daily Living in Hoarding

CHS: Clinically relevant hoarding symptoms

* Chi-square test statistic and p-value from Pearson’s chi-square tests

^a^ significantly different from the ‘CHS only’ group (pairwise chi-square test, p<0.01)

^b^ significantly different from the ‘[medical/psychiatric condition] only’ group (pairwise chi-square test, p<0.01)
